# Proteins with Highly Similar Native Folds Can Show Vastly Dissimilar Folding Behavior When Desolvated**

**DOI:** 10.1002/anie.201306838

**Published:** 2013-11-20

**Authors:** Moritz Schennach, Kathrin Breuker

**Affiliations:** Institut für Organische Chemie and Center for Molecular Biosciences Innsbruck (CMBI), Universität InnsbruckInnrain 80–82, 6020 Innsbruck (Austria)

**Keywords:** electron capture dissociation, gas phase, mass spectrometry, protein, protein folding

## Abstract

Proteins can be exposed to vastly different environments such as the cytosol or membranes, but the delicate balance between external factors and intrinsic determinants of protein structure, stability, and folding is only poorly understood. Here we used electron capture dissociation to study horse and tuna heart Cytochromes *c* in the complete absence of solvent. The significantly different stability of their highly similar native folds after transfer into the gas phase, and their strikingly different folding behavior in the gas phase, can be rationalized on the basis of electrostatic interactions such as salt bridges. In the absence of hydrophobic bonding, protein folding is far slower and more complex than in solution.

Gas phase studies of proteins can further our understanding of the protein folding problem^1^ and are critical to assess the use of mass spectrometry for the structural probing of proteins and their complexes.^2^ For example, electrospray ionization (ESI) MS is used to investigate protein–ligand interactions for drug discovery,^2^ and thought to provide novel insights into protein misfolding diseases.^3^ However, the extent to which protein gas phase structures resemble their solution counterparts remains highly controversial.^4^ In a comprehensive picture of the structural evolution of proteins during and after transfer into the gas phase, it was proposed that dehydration ultimately causes unfolding and subsequent folding into stable gas phase structures that can bear little resemblance to the native fold.4a While the hypothesis of protein denaturation effected by loss of solvent is well supported by experiment,^5^ only very few studies have to date investigated the folding of gaseous proteins,5b, 6 and only two of these included data resolved to the amino acid residue level.5b, 6b

Here we address the question of how proteins behave in the complete absence of solvent. We report site-specific unfolding and folding data for gaseous (Fe^III^)Cytochromes *c* from horse (hh Cyt *c*) and tuna heart (th Cyt *c*), which have similar folds (Figure 1) but differ significantly (18 % sequence variation) in amino acid composition (Table 1).^7^ Cytochromes *c* have been extensively studied in both the gas and condensed phases,^8^ and their folding in solution is fairly well understood.^9^ Folding of gaseous Cyt *c* ions has previously been studied by ion mobility spectrometry (IMS)6c and electron capture dissociation (ECD).6b The IMS study6c indicated folding of hh Cyt *c* ions on a 10 s timescale but provided no structural details, whereas the ECD study6b gave site-specific data, but only for structures probed more than 10 s after unfolding.6b In this study, we used ECD to investigate hh and th Cyt *c* structures shortly (200–400 ms) after desolvation, and to monitor their “early”, site-specific folding kinetics on a timescale of up to 10 s after extensive unfolding by collisional activation. Moreover, we report complementary data from native electron capture dissociation (NECD)^10^ of th Cyt *c*.

**Figure 1:**
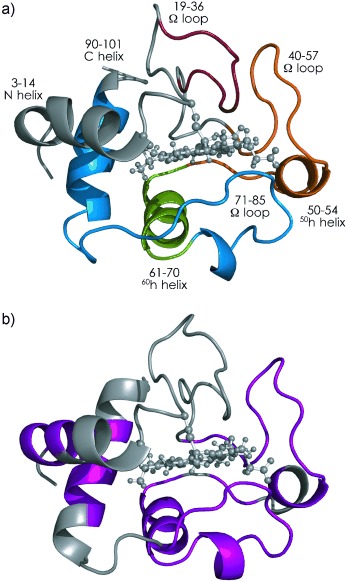
Native folds of a) hh Cyt *c* (pdb entry 1AKK) and b) th Cyt *c* (3CYT); color coding highlights regions defined in Figures 2 and 3.

**Table 1:** Amino acid residue composition of hh and th Cyt *c*

Residues	Type	hh Cyt *c*	th Cyt *c*
H, K, R	basic	24	20
D, E	acidic	12	9
D, E, G, H, K, N, P, Q, R, S, T	polar^[a]^	70	66
A, C, F, I, L, M, V, W, Y	hydrophobic^[a]^	34	37

[a] Classification according to reference 11.

IMS5a and NECD^10^ experiments previously showed that the native fold of hh Cyt *c* (Figure 1 a) disintegrates on a ms timescale after transfer into the gas phase as a result of an essentially reversed order of regional protein stability.5d We find here that the order of stability of sidechain–heme interactions in gaseous th Cyt *c* indicated by NECD, Y46≈T49≈N52>L35>M65≈L68≈K79/M80≈F82>L32≈T78>L98>K13>F10 (see Figure S1 in the Supporting Information), is very similar to that of hh Cyt *c*.5d In both proteins, desolvation first causes separation of the N- and C-terminal helices from the heme group, whereas residues with hydrogen bonding to the heme propionates such as T49 and N52 separate last, consistent with hydrophobic contacts being less stable in the gas phase than electrostatic interactions.5d, 10 However, the NECD data do not provide information about whether or not the α helices (Figure 1) stay intact during the separation process; recent evidence from ECD of the three-helix bundle protein KIX showed that helix structure can be preserved on a timescale of at least 4 s after transfer into the gas phase, provided that the loss of hydrophobic bonding is offset by the strengthening of electrostatic interactions.^12^ Whereas fragments from NECD indicate protein–heme contacts,5d, 10 the *c* and *z*^.^ fragments from ECD indicate the loss of higher-order structure, as they are only observed when not held together by noncovalent bonding.5b, 12

ECD of [*M*+8 H]^9+^ ions from ESI of nondenaturing^13^ solutions now shows that even without vibrational ion activation, the N-terminal helix of hh Cyt *c* (Figure 2 a), and all helices of th Cyt *c* (Figure 3 a), have at least partially unraveled within 400 ms after transfer into the gas phase, as indicated by separated *c, z^.^* fragments from backbone cleavage at site 5 of hh Cyt *c*, and in all helix regions of th Cyt *c*. The data further reveal that the native fold of th Cyt *c* is less stable than that of hh Cyt *c* after transfer into the gas phase as more separated fragments were observed for th Cyt *c* (total yield 12.4 %) than for hh Cyt *c* (4.8 %). This observation is consistent with the smaller number of basic and acidic residues of th Cyt *c* when compared to hh Cyt *c* (Table 1); these residues can form salt bridges that can transiently stabilize the native fold, or parts of it, after desolvation.4a, 12 Finally, the *c, z^.^* fragments from sites 23–42 in Figure 2 a show that the N-terminal 1–22 and the C-terminal 43–104 regions have separated from each other in hh Cyt *c* after 400 ms, although the lack of fragments from cleavage in the 43–102 region indicates partial preservation of native structure and/or the formation of new, higher-order gas phase structure.

**Figure 2:**
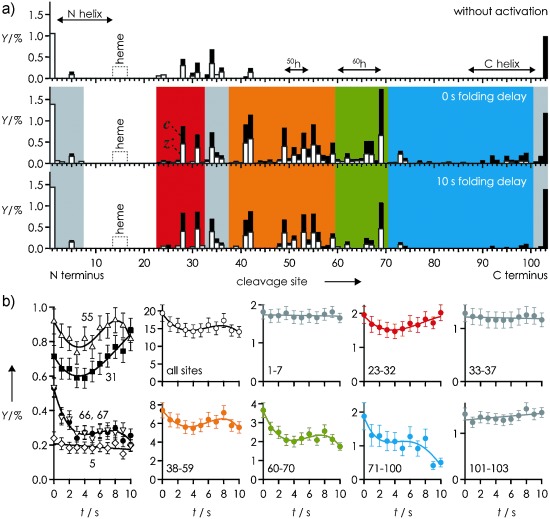
a) *c* (black bars) and *z*^.^ (white bars) fragment yields (*Y*) from ECD of hh Cyt *c* versus cleavage site without (top), and with collisional activation at 0 s (middle) and 10 s (bottom) folding delay; b) *Y* versus folding delay *t* for sites and regions as indicated; see the Supporting Information for error estimation and fit functions.

**Figure 3:**
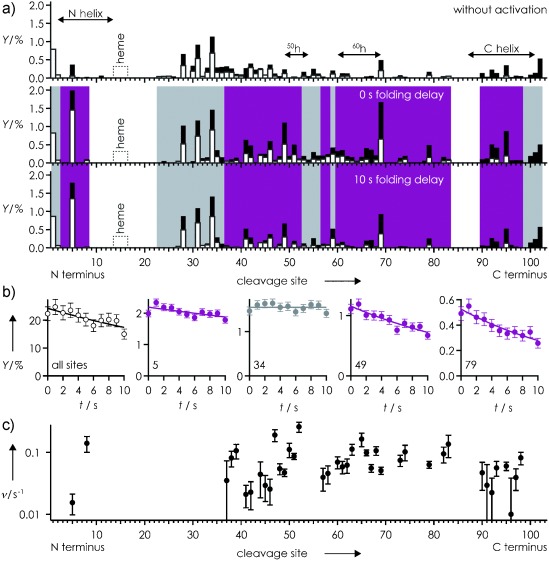
a) *c* (black bars) and *z*^.^ (white bars) fragment yields (*Y*) from ECD of th Cyt *c* versus cleavage site without (top), and with collisional activation at 0 s (middle) and 10 s (bottom) folding delay; b) *Y* versus folding delay *t* for sites as indicated; color coding highlights profiles indicating exponential (violet) or no (gray) folding; c) site-specific rates *ν* from exponential fits, *Y*(*t*)=*Y*_0_ exp(−*ν* *t*), versus cleavage site.

For structure annihilation prior to folding, the [*M*+8 H]^9+^ ions were subjected to 135 eV (laboratory frame energy) collisions with Ar gas, which caused extensive unfolding of the C-terminal 43–104 region of hh Cyt *c* (Figure 2 a), and furthered unfolding of th Cyt *c* (Figure 3 a).

The nonexponential overall folding kinetics of hh Cyt *c* (Figure 2 b, all sites), showing initial folding (0–4 s), unfolding (4–8 s), and again folding (8–10 s), are generally inconsistent with a two-state folding process.^14^ This unusual kinetic profile was observed for most sites (e.g., 55), although the profiles of some sites showed only marginal evidence for unfolding (e.g., 66, 67) or final folding (e.g., 31), and that of other sites (e.g., 5) showed no evidence for folding at all (Figure 2 b). Sites with similar kinetic profiles were grouped into regions, for which site-specific yields were added, and color-coded by type (Figure 2). The data for regions coded in gray (1–7, 33–37, 101–103) showed no significant evidence of folding, which indicates that no noncovalent bonds between the N-terminal 1–37 and the C-terminal 38–104 subdomains were formed on the timescale studied. Nevertheless, the kinetic profiles of the 23–32 (red), 38–59 (orange), 60–70 (green), and 71–100 (blue) regions indicated concerted initial folding. The red region showed continued unfolding, whereas the orange and green regions first showed unfolding and then folding during the last 3 s, and the blue region showed stalled folding. Notably, the propensity for unfolding progressively decreased from the red to the blue region, thereby suggesting that major structural rearrangements during the folding process actually lead to unfolding in the red region, which delays further folding in neighboring regions.

The folding of hh Cyt *c* in the gas phase is completely different from that in solution, where the N-terminal and C-terminal helices (Figure 1) form and associate in an initial step, the ^60^h helix and the 19–36 Ω loop fold next, and the two large 40–57 and 71–85 Ω loops form in a final step.^15^ Although the regions of hh Cyt *c* in Figure 2 a appear to somehow relate to the solution structure (Figure 1 a), its folding behavior implies that the corresponding gas phase structures do, in fact, bear little resemblance to the native fold.4a

The folding of th Cyt *c* in the gas phase (Figure 3 b) is far less complex than that of hh Cyt *c*, despite the fact that their ECD spectra immediately after vibrational ion activation are very similar (Figure 2 a, Figure 3 a). Only two types of kinetic profiles were observed for th Cyt *c* (Figure 3 b), indicating either no or exponential folding, the rates of which (Figure 3 c) differed by more than an order of magnitude. The highest rates (≥0.05 s^−1^) were found for sites 8, 38, 39, 93, 95, 98, and regions 47–52 and 60–83. However, these are separated by sites or regions with kinetic profiles indicating no (23–36, 53–56, 59) or far slower (41–47, 57, 58) folding, which suggests that local interactions between residues neighboring in sequence drive the folding of gaseous th Cyt *c*, in strong contrast to the nonlocal, hydrophobic interactions proposed as driving forces for protein folding in solution.^16^ Moreover, folding of th Cyt *c* in the gas phase is far more uniform than that of hh Cyt *c*, and slower by a factor of approximately 2 during the first approximately 4 s (Figures 2 and 3).

Because ion charge was the same in all ECD experiments, 9+, and the average charge of fragment ions from ECD of unfolded precursor ions showed no significant differences (Figure 4), it is unlikely that charge location is responsible for the strikingly different folding behavior of hh and th Cyt *c*. However, the number of polar residues according to the classification by Dill and co-workers^11^ is somewhat higher in hh Cyt *c*, and that of hydrophobic residues is correspondingly higher in th Cyt *c* (Table 1). More specifically, the integrated index of hydropathy^17^ is −93.8 for hh but only −74.9 for th Cyt *c*, which shows that both proteins are hydrophilic (negative index values), but th Cyt *c* is approximately 20 % less hydrophilic than hh Cyt *c*. The faster initial folding of hh Cyt *c* and its substantially higher polarity when compared to th Cyt *c* strongly suggest that electrostatic interactions such as salt bridges and hydrogen bonds are critically involved in the folding of gaseous proteins.

**Figure 4:**
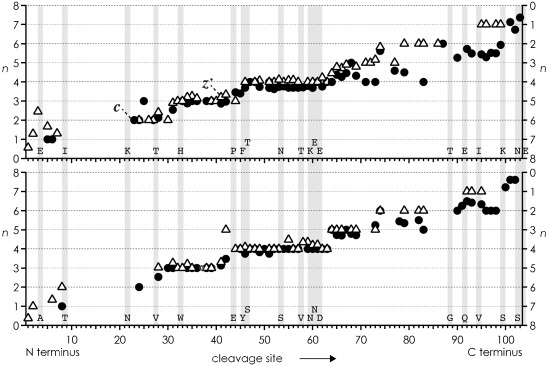
Average charge *n* of *c* (•, left axis) and *z*^.^ (▵, right axis) fragments from ECD of hh (top) and th (bottom) Cyt *c* immediately after collisional activation versus cleavage site; gray bars highlight nonidentical residues.

While salt bridges were recently found to transiently stabilize the three-helix bundle structure of KIX after transfer into the gas phase,^12^ it is yet unclear what role they could play in the folding of gaseous proteins. To address this question, we considered all salt bridges that could potentially form in hh and th Cyt *c* after unfolding (Figure 5). The higher total number of possible salt bridges for hh Cyt *c* (336) compared to that for th Cyt *c* (240), together with the initially faster and then more complex folding of hh Cyt *c*, suggests that salt bridge formation is an important factor in folding in the gas phase. In support of this hypothesis, the density of possible salt bridges was generally high in regions of both hh and th Cyt *c*, the kinetic profiles of which indicated folding (blue, green, orange, violet), whereas regions without evidence for folding (red, gray) generally lacked the possibility for salt bridge formation (Figure 5). Importantly, the blue region, which showed the highest propensity for folding in hh Cyt *c*, and the violet 60–98 region, which showed consistently high folding rates in th Cyt *c*, have the highest number of possible salt bridges, and appear to act as folding nuclei.

**Figure 5:**
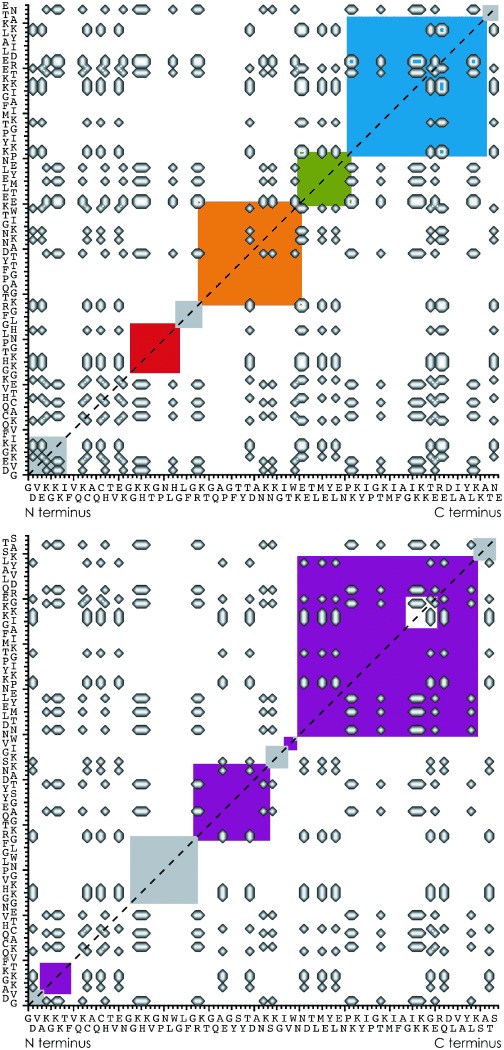
Possible salt bridges between basic (H, K, R) and acidic (D, E, heme propionates, C terminus) residues in hh (top) and th (bottom) Cyt *c* shown as cross peaks; colored squares indicate regions defined in Figures 2 and 3.

Unlike in solution, where Cyt *c* and most other proteins with apparent two-state folding behavior initially fold by formation and association of their N- and C-terminal secondary structural elements,15a the “early” (up to 10 s) folding of fully desolvated Cyt *c* is limited to local interactions between residues neighboring in sequence, without any evidence for contacts between the termini, or the formation of global structure. Moreover, folding of hh Cyt *c* in the gas phase was still incomplete after 120 s.6b These observations are consistent with electrostatic interactions taking over the role of hydrophobic interactions as a major driving force^16^ for protein folding in the gas phase.

In conclusion, we show here that the native folds of hh and th Cyt *c* disintegrate on a timescale of 400 ms after desolvation. Consistent with its smaller number of possible salt bridges, the fold of th Cyt *c* disintegrates faster than that of hh Cyt *c*. Folding in the gas phase of hh and th Cyt *c* after extensive structural annihilation is far slower than Cyt *c* folding in solution,^18^ and shows strikingly different kinetic profiles, despite their almost identical native folds. Once a native fold is lost after desolvation, folding in the gas phase can produce more compact structures, but these bear no or little resemblance to the original fold.

In a discussion of the protein folding problem, Dill and MacCallum posed the important question “How can proteins fold so fast?”1b Our data on protein folding in the gas phase suggest that fast folding, on a μs to ms timescale, or even the formation of structural elements involving approximately 10 or more residues neighboring in sequence, is highly unlikely in the absence of hydrophobic interactions. The search of a gaseous protein for minimum energy structures requires exploring all possible electrostatic interactions without structural restraints from hydrophobic bonding, which can slow the folding process to the extent that transient rather than stable structures may be probed in gas phase experiments on ms timescales. However, gas phase folding of proteins that function in far less polar environments such as membranes might be more similar to their folding in solution, a topic which we plan to address in future studies.

## Experimental Section

Experiments were performed on a 7 T Fourier transform ion cyclotron resonance (FT-ICR) mass spectrometer (Bruker, Austria) equipped with an ESI source and a hollow dispenser cathode for ECD. Cytochromes *c* from horse and tuna heart (Sigma Aldrich, Austria; horse: GDVEKGKKIF VQKCAQCHTV EKGGKHKTGP NLHGLFGRKT GQAPGFTYTD ANKNKGITWK EETLMEYLEN PKKYIPGTKM IFAGIKKKTE REDLIAYLKK ATNE; tuna: GDVAKGKKTF VQKCAQCHTV ENGGKHKVGP NLWGLFGRKT GQAEGYSYTD ANKSKGIVWN NDTLMEYLEN PKKYIPGTKM IFAGIKKKGE RQDLVAYLKS ATS) were electrosprayed from 3 μm solutions (95:5 H_2_O/CH_3_OH at pH 4.2, adjusted by addition of CH_3_COOH). ESI source and ion transfer conditions were tuned to minimize undesired vibrational ion activation by collisions with background gas. [*M*+8 H]^9+^ ions were accumulated in the first hexapole for 0.2 s, isolated by *m*/*z* in the quadrupole, accumulated in the second hexapole for 0.2 s, and transferred (2.5 ms) into the trapped ICR cell for ECD (25 ms, <0.5 eV electron energy) and ion detection. For unfolding, protein ions were vibrationally activated by energetic collisions with Ar gas at the head of the second hexapole. Subsequent folding was monitored by varying the delay between ion accumulation in the second hexapole and transfer into the ICR cell from 0–10 s in 1 s intervals. Between 250 and 500 scans were added for each spectrum. Site-specific fragment yields were calculated as % values relative to all ECD products, considering that backbone cleavage gives a pair of complementary *c* and *z*^.^ ions (*a*^.^, *y* ions were not included in the analysis because of their marginal abundance totaling to <1 %): 100 %=0.5 [*c*]+0.5 [*z*^.^]+[reduced molecular ions and loss of small neutral species from the latter].

## Supplementary material

As a service to our authors and readers, this journal provides supporting information supplied by the authors. Such materials are peer reviewed and may be re-organized for online delivery, but are not copy-edited or typeset. Technical support issues arising from supporting information (other than missing files) should be addressed to the authors.
