# Supplementary material for: Proteins with Highly Similar Native Folds Can Show Vastly Dissimilar Folding Behavior When Desolvated
Source: Angew Chem Int Ed Engl. 2013 Nov 20;53(1):164–8. doi: 10.1002/anie.201306838 (PMC4065370; doi:10.1002/anie.201306838)
Supplement: Supplementary file 1 [file anie0053-0164-SD1.pdf]

Angewandte Chemie
Eine Zeitschrift der Gesellschaft Deutscher Chemiker

Supporting Information

© Wiley-VCH 2013

69451 Weinheim, Germany

Proteins with Highly Similar Native Folds Can Show Vastly Dissimilar Folding Behavior When Desolvated**

*Moritz Schennach and Kathrin Breuker**

anie_201306838_sm_miscellaneous_information.pdf

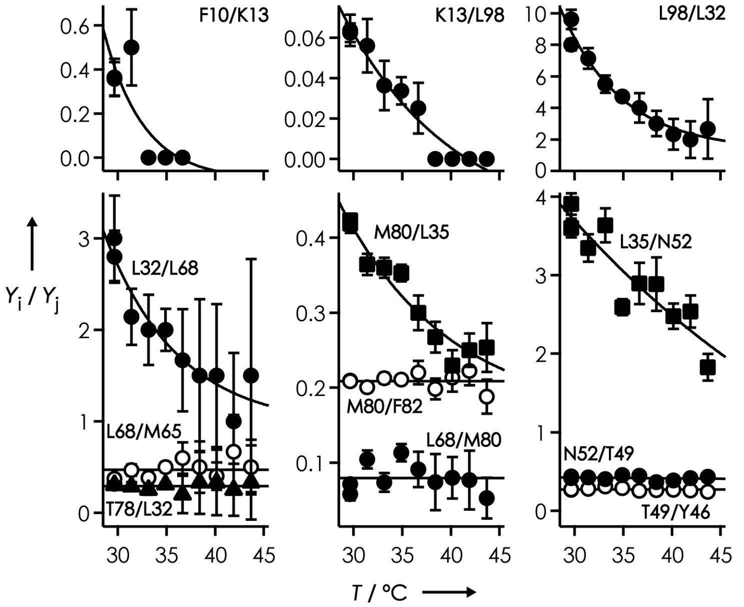

Figure S1. Branching ratio of NECD fragment ions from protein backbone cleavage at sites i and j, (Y_i/Y_j), as indicated. For NECD, th Cyt *c* was dissolved in water (18 MΩ·cm) to a final concentration of 65 μM, the pH adjusted to 4 by addition of acetic acid, and the solution was stored at 4 °C for 6 months. ESI utilized homemade emitters with a tip inner diameter of ~5 μm, flow rate 200–500 nLmin^{-1}, and 1 kV spray potential. Temperature was measured at the capillary orifice through which ions enter the mass spectrometer. The NECD experiments were performed on a 6 T FT-ICR instrument as described in references 4d and 9.

Error estimation for fragment ion yield data in Figures 2b and 3b

Figures 2b and 3d show fragment ion yields derived from ECD spectra that were averaged over 250 to 500 individual scans to minimize statistical errors. Nevertheless, the data for th Cyt *c* in Figure 3b show statistical deviation from exponential behavior; representative data are shown in Figures S2a,b. The data for hh Cyt *c* are better described by polynomial fit functions (see Figure S3). For error estimation, residuals *R* were calculated from exponential fit functions ($Y(t) = Y_0 \cdot \exp(-\nu \cdot t)$; rates ν are shown in Figure 3c) of site-specific fragment yields *Y* from ECD of th Cyt *c* (Figure S2). Statistical analysis of the residuals *R* gave standard deviations σ for each cleavage site. Figure S2c shows standard deviations σ versus average yield values of the fit functions (which is justified as the fit functions are nearly linear within the 0-10 s range considered here). As expected for a counting experiment, the data in Figure S2c can be described by a square-root function, $\sigma(Y) = A \cdot \mathrm{Sqrt}(Y)$, with A = 0.075534 ± 0.00298 (Figure S2c). Site-specific errors in Figures 2b and 3b were calculated from this function, $\sigma(Y) = 0.075534 \cdot \mathrm{Sqrt}(Y)$. Error bars for added yields (error bars for regions in Figure 2b) were calculated by addition of individual errors.

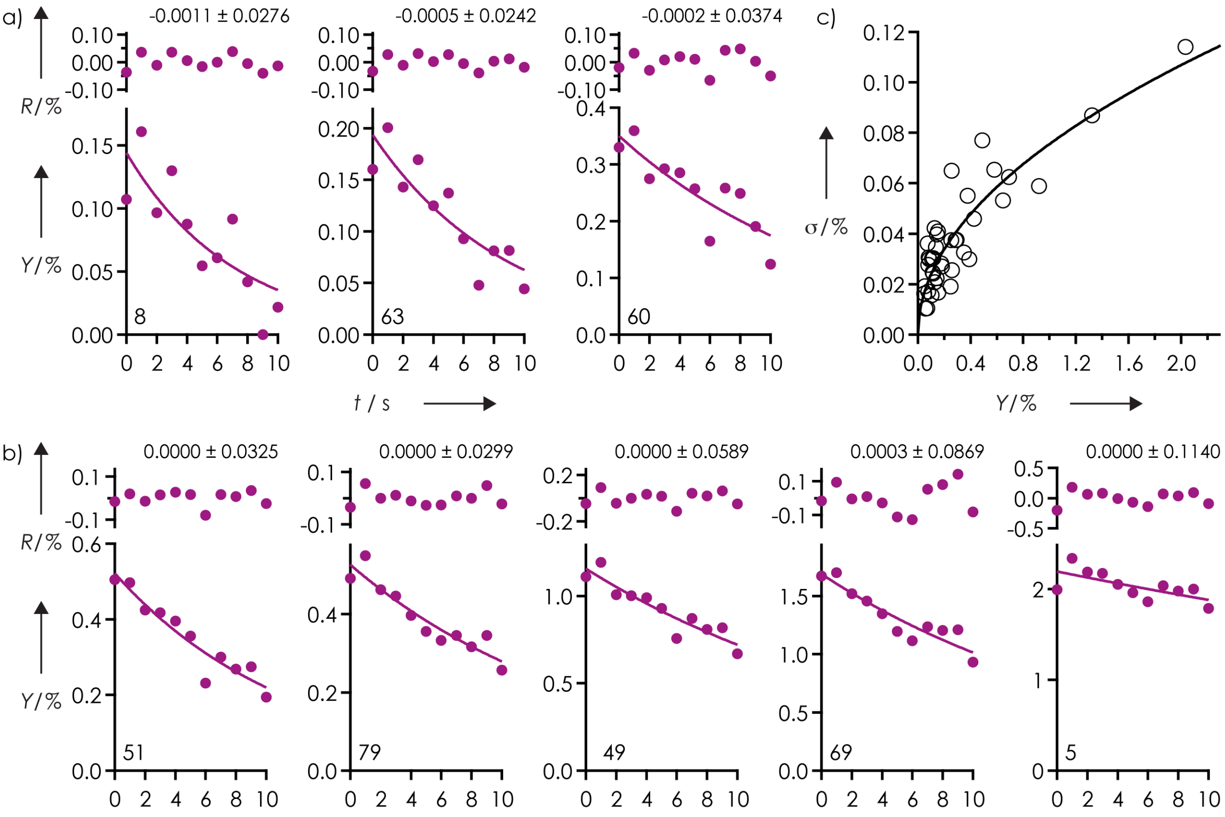

Figure S2. a, b) Exponential fitting (lines) of site-specific fragment yields *Y* (circles) from ECD of th Cyt *c* $(M + 8H)^{9+}$ ions (Figure 3b) gave residuals *R*; statistical analysis of the residuals gave mean values and standard deviations σ indicated above each plot (mean value ± σ); c) standard deviation versus average yield shown as circles, the line shows the fit function $\sigma(Y) = 0.075534 \cdot \mathrm{Sqrt}(Y)$.

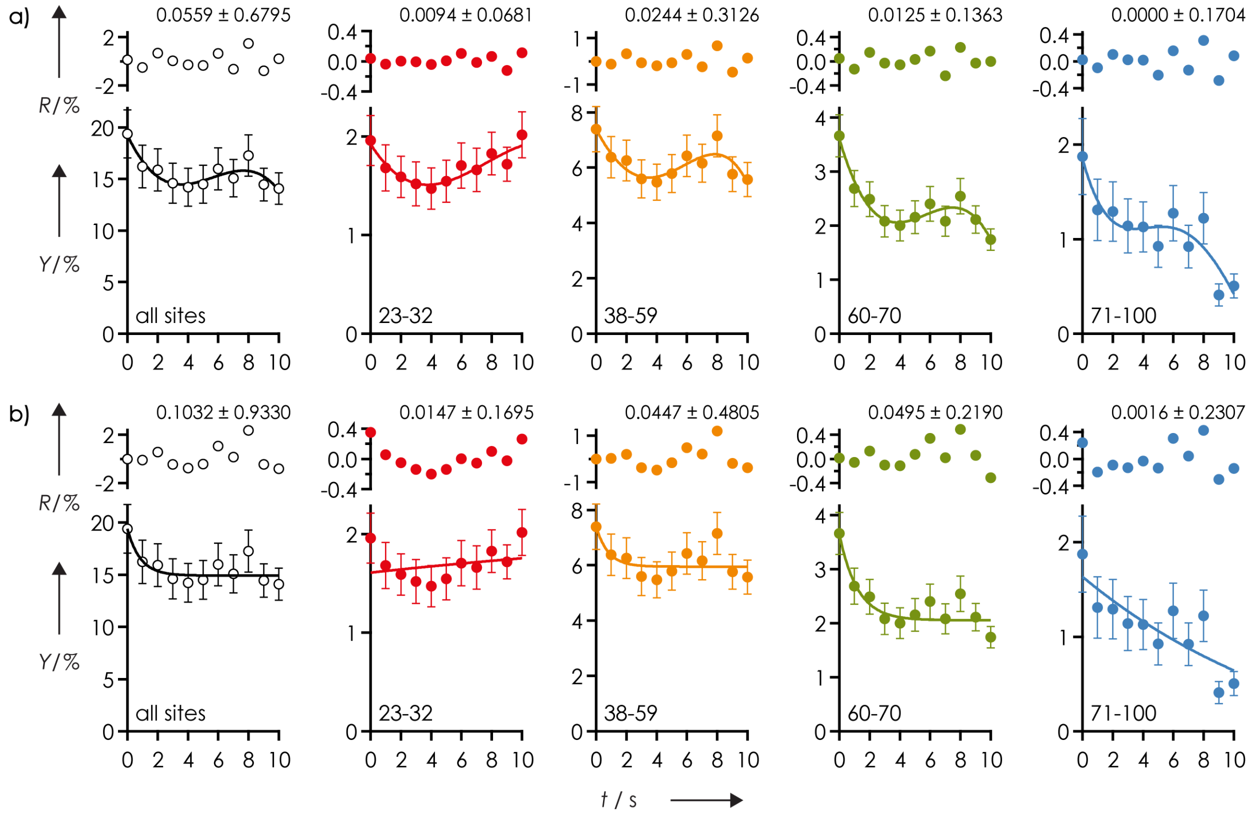

Figure S3. Polynomial (a) vs. exponential (b) fitting of fragment yield (*Y*) data from Figure 2 (ECD of hh Cyt *c* (M + 8H)$^{9+}$ ions) gave residuals *R* whose statistics (mean value ± standard deviation, indicated above each plot) show that polynomial fit functions describe the data significantly better than exponential fit functions. The polynomial fit functions were chosen to illustrate the complex folding behavior of hh Cyt *c*, and are not intended to imply a specific folding model.
